# Regional variations in corneal oedema during open‐eye fenestrated scleral lens wear

**DOI:** 10.1111/opo.13489

**Published:** 2025-03-13

**Authors:** Asif Iqbal, Damien Fisher, David Alonso‐Caneiro, Michael J. Collins, Stephen J. Vincent

**Affiliations:** ^1^ Contact Lens and Visual Optics Laboratory, Optometry and Vision Science, Centre for Vision and Eye Research Queensland University of Technology Brisbane Queensland Australia; ^2^ School of Science, Technology and Engineering University of Sunshine Coast Petrie Queensland Australia

**Keywords:** corneal oedema, fluid reservoir, lens fenestration, scleral lens, tear exchange

## Abstract

**Purpose:**

To compare the magnitude of central, mid‐peripheral and peripheral stromal corneal oedema induced during short‐term fenestrated and non‐fenestrated scleral lens wear.

**Methods:**

Nine healthy participants wore a non‐fenestrated and a fenestrated (0.3‐mm diameter limbal fenestration) scleral lens (KATT™, Capricornia Contact Lenses), hexafocon B material (Dk 141 × 10^−11^ cm^3^ O_2_(cm)/[(s) (cm^2^) (mmHg)]) in one eye under open‐eye conditions for 90 min on two separate days. Scleral lens thickness, fluid reservoir thickness and stromal corneal oedema were measured using high‐resolution optical coherence tomography. Stromal oedema was quantified across the central (0–2.5 mm from the corneal apex), mid‐peripheral (−3.0 to −1.0 mm from the scleral spur) and peripheral (−1.0 to 0 mm from the scleral spur) cornea with the lens in situ. The magnitude of oedema was corrected based on variations in fluid reservoir thickness between the lens conditions.

**Results:**

There was a significant effect of lens type (*p* = 0.04) on stromal oedema, with less oedema observed with the fenestrated (0.36 ± 0.45%) compared to the non‐fenestrated lenses (1.24 ± 0.27%), averaged across all corneal locations. A significant lens type by corneal location interaction was also observed (*p* = 0.05), with less oedema observed in the peripheral region for the fenestrated (−0.15 ± 0.98%) compared to the non‐fenestrated lenses (1.81 ± 0.57%) (*p* = 0.048). A fenestration location by corneal location interaction was also observed (*p* = 0.02), indicating a greater reduction in oedema closer to the fenestration.

**Conclusions:**

Central and mid‐peripheral stromal oedema was similar during fenestrated and non‐fenestrated lens wear; however, fenestrated lenses displayed significantly less oedema in the peripheral cornea. This is most likely due to increased oxygen delivery in proximity to the fenestration.

**Key points**

- A single peripheral fenestration can significantly reduce scleral lens‐induced peripheral corneal swelling, which may be beneficial for susceptible post‐surgical corneas.
- Future research is required to understand the mechanism underlying enhanced localised oxygen supply with a fenestration, such as enhanced tear exchange or passive oxygen diffusion.
- Fenestrated scleral lenses may also enhance patient comfort and ease of lens removal by reducing suction forces, while also addressing issues such as midday fogging and conjunctival compression.

## INTRODUCTION

Fenestrations have been incorporated into soft,^1^, ^2^ corneal rigid^3^, ^4^ and scleral contact lenses^5^, ^6^, ^7^, ^8^ in an attempt to enhance tear exchange and reduce corneal oedema with varying degrees of success. While modern, highly oxygen‐permeable non‐fenestrated scleral lenses typically only induce ~1%–3% central corneal oedema on average in healthy^9^, ^10^, ^11^, ^12^ and keratoconic eyes,^13^, ^14^, ^15^ greater levels of scleral lens‐induced oedema have been reported in eyes that have undergone penetrating keratoplasty (up to ~5%)^16^ and radial keratotomy (up to ~7%).^17^ In such cases, scleral lens modifications such as fenestrations or channels may potentially be beneficial to increase tear exchange, facilitate the release of carbon dioxide from the fluid reservoir, and decrease lens suction.^18^

A number of early clinical studies reported that fenestrations delayed the onset and intensity of Sattler's veil during low oxygen permeable scleral lens wear (glass or polymethylmethacrylate materials), suggesting a reduction in corneal oedema based on patient feedback.^5^, ^6^ Ko et al.^7^ measured tear exchange in both fenestrated and channelled scleral lenses and concluded that, even with such lens modifications, the rate of tear exchange fell below the theoretical metabolic requirements of the cornea and questioned the clinical value of fenestrations for improving oxygen delivery. To date, only one study has quantified the effect of a scleral lens fenestration upon corneal oedema.^8^ This investigation found that a single peripheral fenestration in a highly oxygen permeable material had minimal impact upon central corneal oedema in healthy eyes (fenestrated, 0.50%; non‐fenestrated, 0.62%) or a 19% relative reduction in central swelling. However, the potential effect upon peripheral corneal oedema was not examined.

Given previous reports of a reduction in corneal hypoxic stress localised to the fenestration position in corneal rigid lens wear,^19^, ^20^ regional variations in corneal oedema may also occur during fenestrated scleral lens wear. Therefore, the aim of the study was to examine the effect of a single peripheral fenestration on central, mid‐peripheral and peripheral corneal oedema during short‐term open‐eye scleral lens wear in comparison to a non‐fenestrated scleral lens control condition.

## METHODS

This study was approved by the Queensland University of Technology human ethics research committee and followed the tenets of the Declaration of Helsinki. Following an explanation regarding the nature of the experiment, informed consent was obtained from all participants. The protocol for this experiment has been published previously.^8^

### Participants

Nine participants (mean age (standard error): 30 (1) years) with healthy eyes and visual acuity of 0.00 logMAR or better in both eyes were recruited. A preliminary ophthalmic screening was carried out to exclude potential participants exhibiting any significant ocular or vision abnormality, a history of ocular injury or surgery, contraindications to contact lens wear, current use of topical medication or regular rigid contact lens wear. Participants who habitually wore soft contact lenses (*n* = 5) were instructed to discontinue lens wear at least 24 h preceding each experimental session.

### Scleral lens design and fitting

Following the initial screening, the left eye of each participant was fitted with scleral lenses (KATT™, Capricornia Contact Lenses, capcl.com.au) manufactured in hexafocon B material (Dk 141 × 10^−11^ cm^3^ O_2_(cm)/[(s) (cm^2^) (mmHg)]), with a back vertex power of −1.00 D, a total diameter of 16.5 mm with a spherical landing zone, a nominal central lens thickness of 300 μm and a back optic zone radius of 7.46 mm. The fenestrated and non‐fenestrated lenses were manufactured with the same specifications except the addition of one 0.3‐mm diameter fenestration located 6.25 mm from the centre of the optic zone (2 mm from the edge of the lens, approximately overlying the limbus). The thickness of each scleral lens across the central 12.5 mm was quantified using a technique described previously.^21^

The initial scleral lens was selected based on the average corneal sagittal height measured over a 10‐mm chord with a videokeratoscope (E300; Medmont, medmont.com.au). An additional 2000 μm was added to this corneal sagittal height to extrapolate to a 15‐mm chord,^22^ and an additional value was added to achieve the desired central initial fluid reservoir thickness of 150 μm. The lens was then applied with preservative‐free saline (Lens Plus Ocupure, Abbott Medical Optics, abbott.com) and the central fluid reservoir thickness was measured at the corneal apex using the in‐built callipers of an optical coherence tomographer (OCT, Spectralis, Heidelberg Engineering, heidelbergengineering.com).

### Experimental details and image analysis

Following the initial screening, participants returned to the laboratory on two different occasions for fenestrated and non‐fenestrated lens wear in a randomised order. The sessions were separated by at least 24 h and conducted at the same time of day, at least 2 h after waking. Central and peripheral anterior segment OCT scans were obtained immediately after lens application and again after 90 min of lens wear prior to lens removal. Central scans were centred on the pupil, and nasal and temporal peripheral scans were obtained while participants maintained fixation on an external target positioned 26.5 degrees laterally. A volumetric scanning protocol was used (3 × ~8 mm horizontal line scans consisting of 20 B‐scans, separated vertically by 139 μm). OCT images were exported for analysis using customised software. The software segmented the posterior scleral lens surface, anterior epithelium, anterior stroma and endothelium. Any segmentation errors were manually corrected if required. Three measurements were averaged for each participant at each time point for both lens types. Central thickness measurements were constrained to the central 5 mm (0–2.5 mm from the corneal apex). Mid‐peripheral (−3.0 to −1.0 mm) and peripheral (−1.0 to 0 mm) corneal thickness measurements were referenced to the location of the scleral spur (0 mm). The measurements were averaged across nasal and temporal sides for both fenestrated and non‐fenestrated lenses (except for one analysis examining the nasal cornea alone in three participants where the fenestration was positioned nasally along the horizontal meridian). Three OCT line scans from a single image were averaged for each time point. This image segmentation and analysis procedure has been described previously.^23^ Scheimpflug imaging (Pentacam HR, pentacam.com) was also undertaken following the OCT measurements at baseline and prior to lens removal after 90 min of lens wear using the 50 scan mode (i.e., one radial scan approximately every 7 degrees). The Scheimpflug images were used to visualise the location of the scleral lens fenestration only, and no other data were extracted from the Pentacam instrument.

### Correction for variations in initial fluid reservoir thickness

Due to differences in the initial fluid reservoir thickness between the fenestrated and non‐fenestrated lens conditions, particularly in the mid‐periphery and the periphery, the stromal corneal oedema data were corrected using measurements from previous experiments which quantified the magnitude of central, mid‐peripheral and peripheral corneal oedema during open‐eye non‐fenestrated scleral lens wear as a function of fluid reservoir thickness for the same participants.^23^ Polynomial (for central data) and linear (for mid‐peripheral and peripheral data) equations were derived for each participant from these previous experiments which quantify the relationship between the corneal oedema as a function of fluid reservoir thickness. These equations were then used to estimate the magnitude of corneal oedema due to a difference in fluid reservoir thickness between the fenestrated and non‐fenestrated conditions. The measured corneal oedema was then corrected to account for this by subtracting this value from the oedema value of the condition with the greater fluid reservoir thickness. This approach has been described in detail previously.^10^ No corrections were made for differences in lens thickness (which were typically <15 μm) because no studies to date have examined the effect of altering peripheral lens thickness upon corneal oedema, and such differences in lens thickness only result in very small variations in central oedema for a material with a Dk of 141 under open‐eye conditions (~0.02%).^10^

### Statistical analyses

Statistical analyses were performed using SPSS (ibm.com). The required sample size was determined using G*Power (Version 3.1.9.2; Heinrich Heine Universität Dusseldorf, psychologie.hhu.de), assuming an effect size of 1.17 based on the open‐eye central corneal oedema data from a corneal rigid lens study (material Dk 100) comparing non‐fenestrated and fenestrated lenses (one 0.25‐mm fenestration).^24^ For a two‐tailed, matched pairs *t*‐test, assuming a 0.5 correlation between groups, with a type I error probability of 0.05, a sample size of eight participants would provide a power of 0.8 (actual power of 0.81 was achieved based on the experimental results with a sample size of nine for central corneal oedema).

The normality of the data was confirmed using the Kolmogorov–Smirnov test. Stromal oedema data were examined using a series of repeated measures analysis of variance (RM‐ANOVA). The first included two within‐subject factors of lens type (fenestrated and non‐fenestrated) and corneal location (central, mid‐peripheral and peripheral) and their interaction for all participants (*n* = 9). An additional RM‐ANOVA considering the fenestrated lens wear condition only included a within‐subject factor of corneal location (central, mid‐peripheral and peripheral) and a between‐subject factor of fenestration orientation (horizontal or non‐horizontal) and their interaction for participants where the location of the fenestration could be verified in Scheimpflug images (*n* = 7).

Lens thickness and fluid reservoir thickness values were also compared across the three locations (central, mid‐peripheral and peripheral) averaged across nasal and temporal sides for fenestrated and non‐fenestrated lens types using a RM‐ANOVA. Significant main effects or interactions were explored using Bonferroni‐corrected pairwise comparisons. Data are presented as the mean and the standard error. *p*‐values <0.05 were considered statistically significant.

## RESULTS

### Lens thickness

**FIGURE 1:**
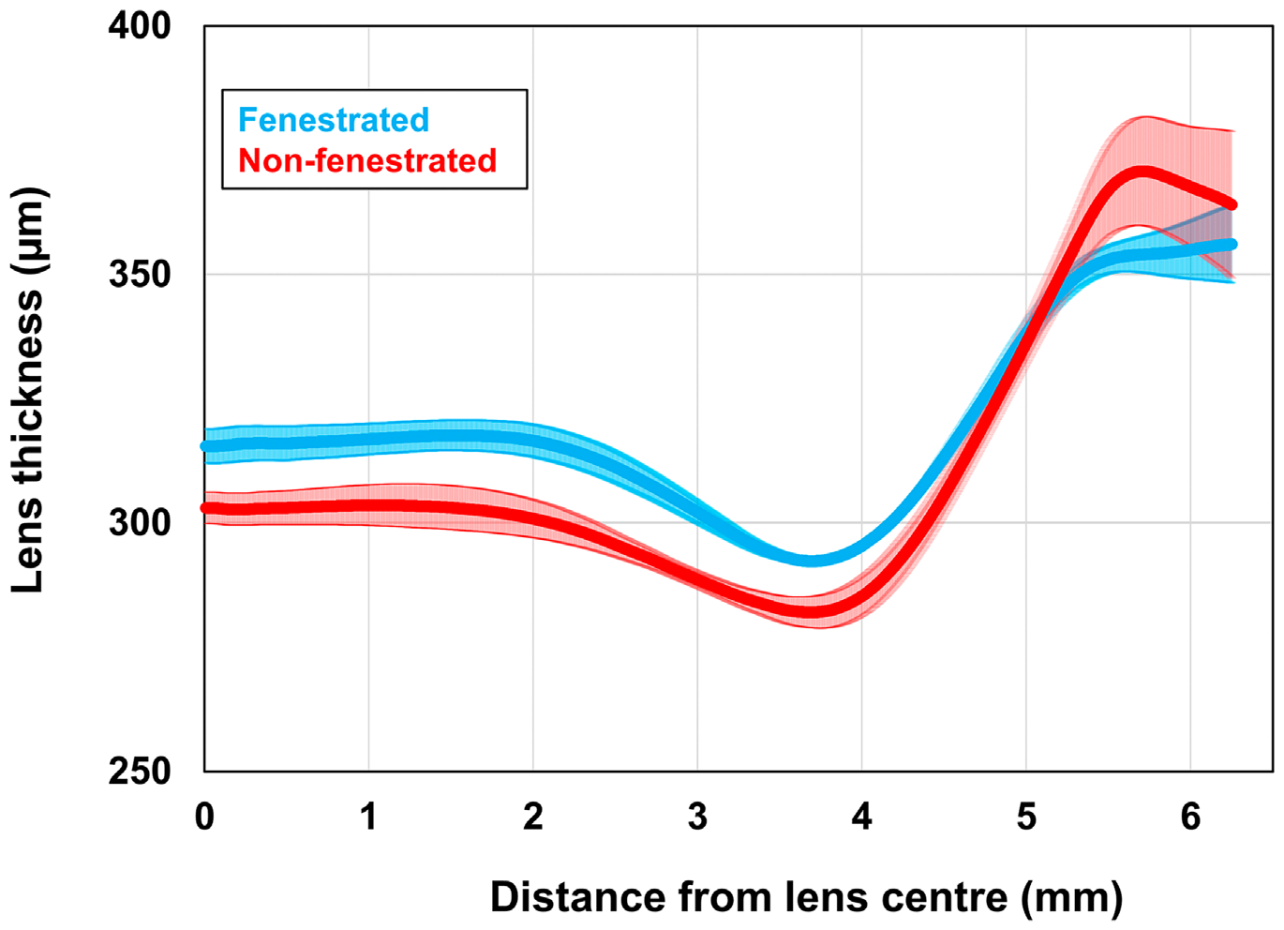
The mean thickness profile for the fenestrated (blue) and non‐fenestrated (red) lenses from centre to periphery. The shaded areas represent the standard error.

Averaged across the entire lens (lens centre to periphery hemi‐chord), there was no significant difference in thickness between fenestrated (324 ± 8 μm) and non‐fenestrated (319 ± 5 μm) lenses (*F*_1,8_ = 0.6, *p* = 0.47) (Figure 1). There was a significant lens by location interaction (*F*_2,16_ = 777, *p* < 0.001) indicating that the fenestrated lenses were slightly thicker in the mid‐periphery compared to the non‐fenestrated lenses (10 ± 12 μm thicker, *p* = 0.03) (Table 1). However, these small differences (<15 μm) across the three locations are not likely to impact corneal oedema significantly.

**TABLE 1:** The mean ± standard error lens thickness (μm) for the fenestrated and non‐fenestrated lenses and results of the repeated‐measures analysis of variance (RM‐ANOVA). Central (0–2.5 mm from lens centre), mid‐peripheral (2.5–5 mm from lens centre) and peripheral (5–6 mm from lens centre).

Location	Lens		RM‐ANOVA
	Non‐fenestrated (μm)	Fenestrated (μm)	Lens × location, *p*‐value
Central	302 ± 4	316 ± 3	0.05
Mid‐peripheral	295 ± 3	305 ± 1	0.03
Peripheral	361 ± 9	351 ± 3	0.29

### Fluid reservoir thickness

**FIGURE 2:**
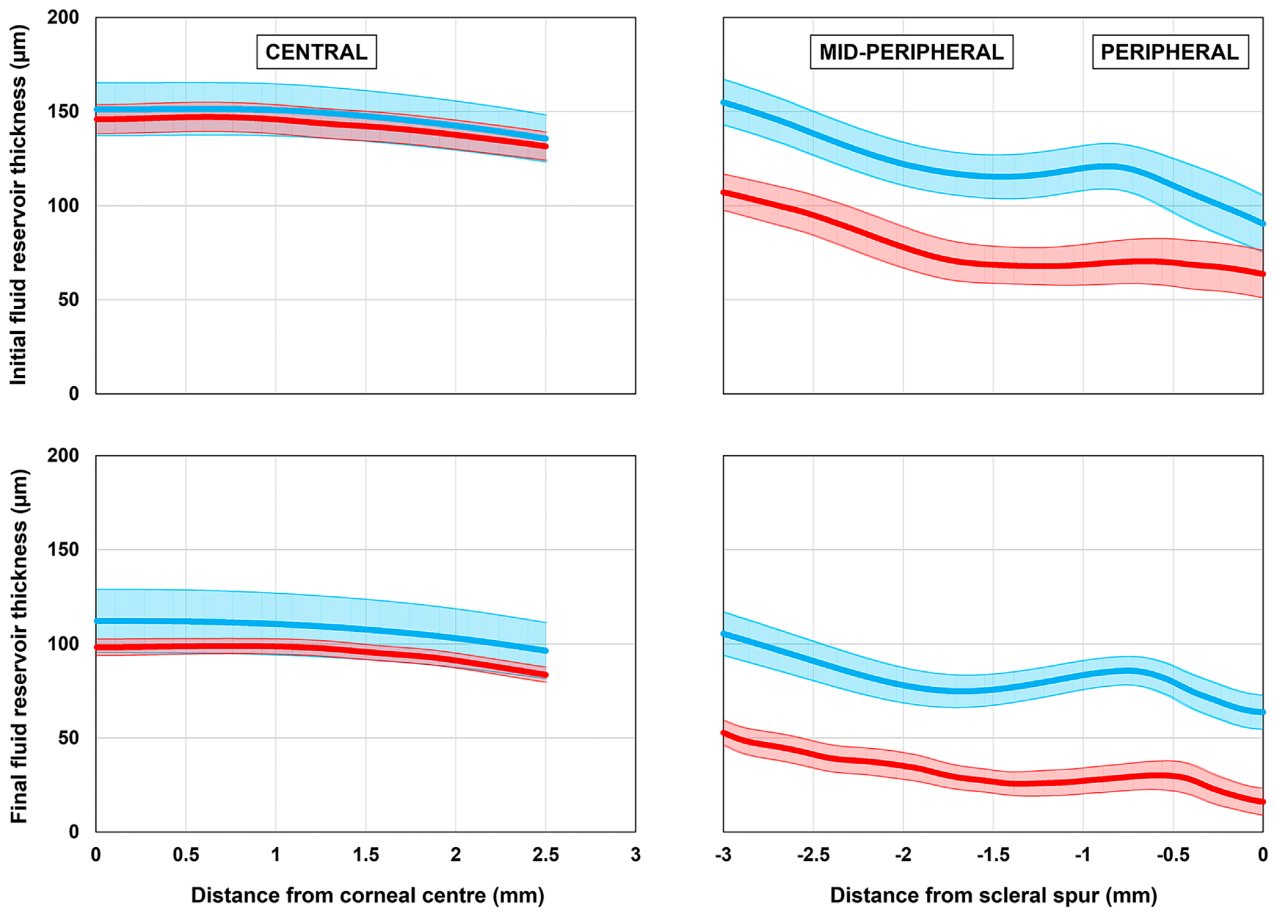
The mean initial (top panel) and final (lower panel) fluid reservoir thickness profile (averaged across nasal and temporal locations) for the fenestrated (blue) and non‐fenestrated (red) lenses for the central, mid‐peripheral and peripheral locations. The shaded areas represent the standard error.

RM‐ANOVA revealed a significant lens type by location interaction for fluid reservoir thickness measures after the initial lens application (*F*_2,16_ = 8.9, *p* = 0.002) and after 90 min of lens wear (*F*_2,16_ = 7.5, *p* = 0.005) (Figure 2). Post hoc comparisons indicated that the fenestrated lenses had a significantly higher fluid reservoir thickness for both mid‐peripheral and peripheral locations compared to the non‐fenestrated lenses (Table 2).

**TABLE 2:** The mean ± standard error initial, final and change in central (0–2.5 mm from the corneal apex), mid‐peripheral (−3.0 to −1.0 mm from the scleral spur) and peripheral (−1.0 to 0 mm from the scleral spur) fluid reservoir thickness (μm) for non‐fenestrated and fenestrated lenses. RM‐ANOVA, repeated‐measures analysis of variance.

Location	Time	Lens		RM‐ANOVA
		Non‐fenestrated (μm)	Fenestrated (μm)	Lens (post hoc *p*‐value)
Central	Initial	142 ± 8	147 ± 13	0.79
	Final	95 ± 4	108 ± 16	0.47
	Change	47 ± 8	40 ± 7	0.26
Mid‐peripheral	Initial	82 ± 10	128 ± 11	0.02
	Final	35 ± 6	84 ± 9	0.001
	Change	47 ± 8	44 ± 5	0.72
Peripheral	Initial	68 ± 12	109 ± 14	0.009
	Final	26 ± 7	77 ± 8	<0.001
	Change	43 ± 8	32 ± 8	0.22

### Stromal oedema

#### Lens type

**FIGURE 3:**
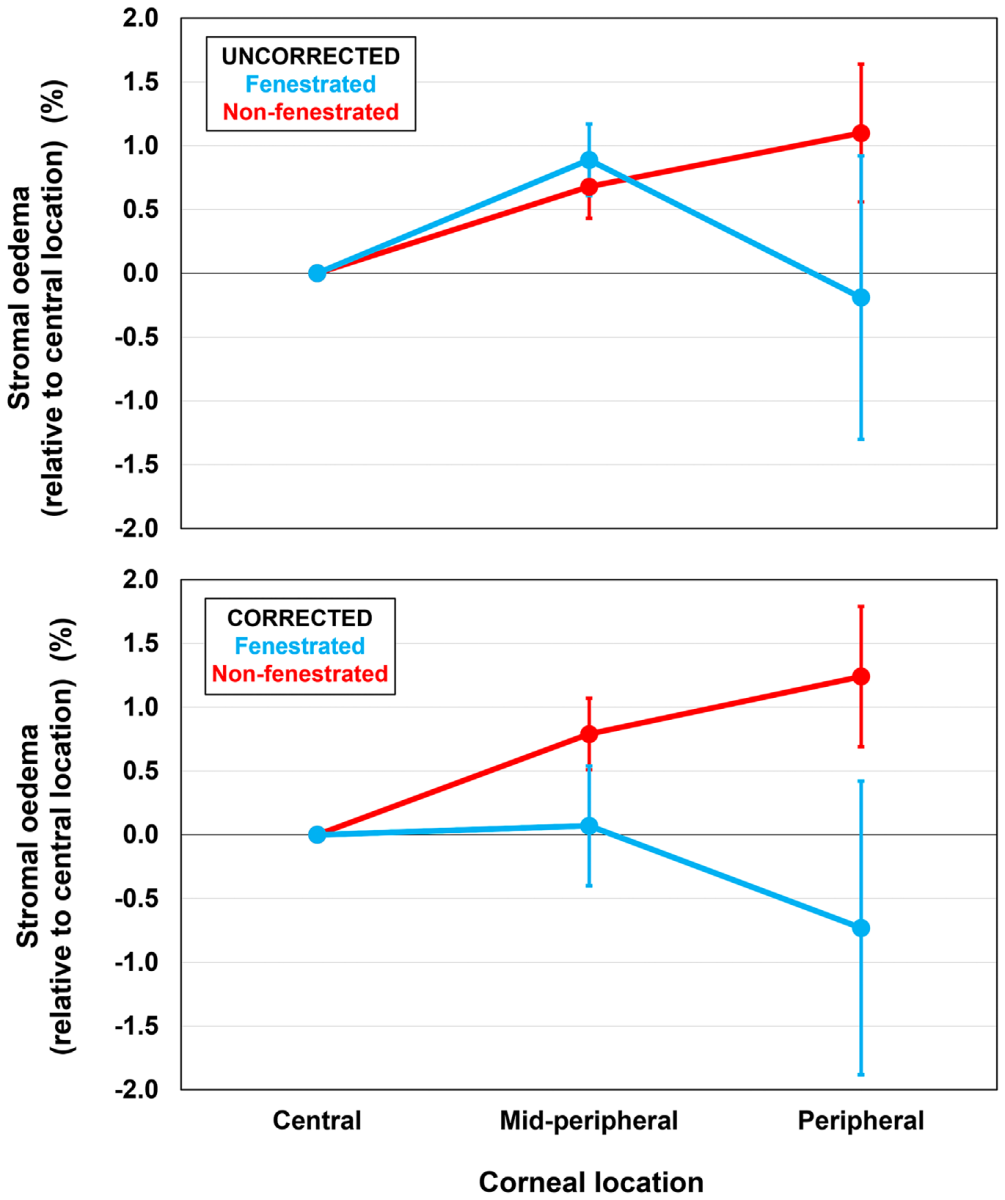
The mean mid‐peripheral and peripheral stromal oedema (%) (averaged across nasal and temporal locations) relative to the central location for fenestrated (blue) and non‐fenestrated (red) lenses. Top panel: Uncorrected for differences in fluid reservoir thickness. Lower panel: Corrected for differences in fluid reservoir thickness. Error bars are the standard error.

**FIGURE 4:**
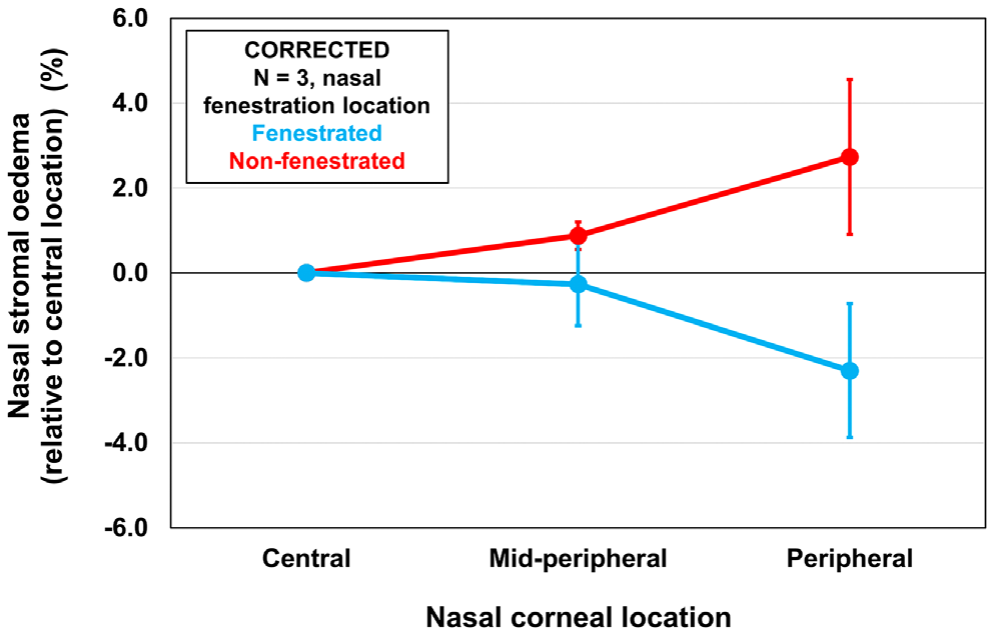
The mean mid‐peripheral and peripheral stromal oedema (%) (averaged across nasal corneal locations only) relative to the central location for fenestrated (blue) and non‐fenestrated (red) lenses in three participants with the fenestration positioned nasally. Error bars are the standard error. Oedema data have been corrected for differences in fluid reservoir thickness.

There was no significant difference in the magnitude of corrected stromal corneal oedema after 90 min of lens wear across corneal locations (*F*_2,16_ = 0.21, *p* = 0.82) when averaged across lens types. There was a significant effect of lens type (*F*_1,8_ = 5.69, *p* = 0.04), with less oedema observed with the fenestrated lenses (0.36 ± 0.45%) compared to non‐fenestrated lenses (1.24 ± 0.27%), averaged across all corneal locations. A lens type by corneal location interaction was also observed (*F*_2,16_ = 3.58, *p* = 0.05), with less oedema being observed in the peripheral region for the fenestrated (−0.15 ± 0.98%) compared to non‐fenestrated lenses (1.81 ± 0.57%) (*p* = 0.048) (Figure 3, Table 3). Figure 4 displays the corrected nasal stromal oedema for three participants where the fenestration was located at the nasal limbus (aligned with the orientation of the OCT scan). This further highlights the effect of a peripheral fenestration, with a relative reduction of more than 4% swelling in this peripheral corneal region.

**TABLE 3:** Mean ± standard error corrected stromal oedema (%) after 90 min of non‐fenestrated (Non) and fenestrated (Fen) lens wear, for the central (0–2.5 mm from the corneal apex), mid‐peripheral (−3.0 to −1.0 mm from the scleral spur) and peripheral (−1.0 to 0 mm from the scleral spur) locations. RM‐ANOVA, repeated‐measures analysis of variance.

Location	Lens		RM‐ANOVA
	Non‐fenestrated (%)	Fenestrated (%)	*p*‐value
Central	0.56 ± 0.15	0.57 ± 0.37	Lens × location: 3.58 (0.05) Post‐hoc comparison: Peripheral: Fen < Non (0.048)
Mid‐peripheral	1.35 ± 0.38	0.65 ± 0.63	
Peripheral	1.81 ± 0.57	−0.15 ± 0.98	

#### Fenestration orientation

**FIGURE 5:**
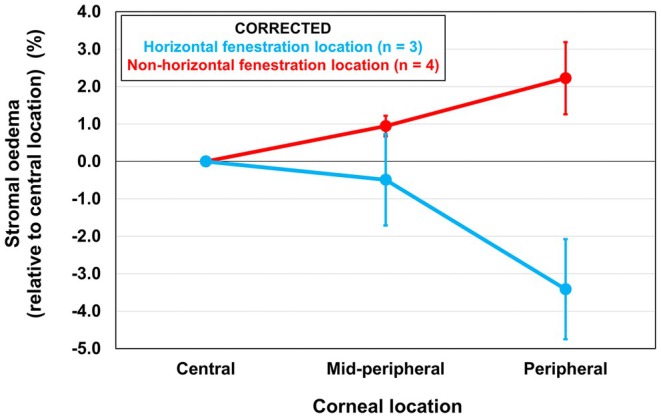
The mean mid‐peripheral and peripheral stromal oedema (%) (averaged across nasal and temporal locations) relative to the central location for fenestrated lenses only, with the fenestration located along the horizontal meridian (blue) and non‐horizontal meridians (red). Error bars are the standard error. Oedema data have been corrected for differences in fluid reservoir thickness.

RM ANOVA revealed a significant corneal location by fenestration orientation interaction (*F*_2,10_ = 5.81, *p* = 0.02) (Figure 5). Less relative corneal oedema was observed in the peripheral cornea (along the horizontal meridian, where OCT scans were captured) when the fenestration was oriented along the horizontal meridian (−2.92 ± 0.63%) compared to when the fenestration was oriented in a non‐horizontal meridian (2.49 ± 1.03%; *p* = 0.01; Table 4).

**TABLE 4:** Mean ± standard error corrected stromal oedema after 90 min of fenestrated lens wear (averaged across nasal and temporal locations) for participants with the fenestration located along the horizontal (*n* = 3) and non‐horizontal (*n* = 4) meridians. The locations refer to the central (0–2.5 mm from the corneal apex), mid‐peripheral (−3.0 to −1.0 mm from the scleral spur) and peripheral (−1.0 to 0 mm from the scleral spur) cornea. RM‐ANOVA, repeated‐measures analysis of variance. H, Horizontal fenestration orientation; N, Non‐horizontal fenestration orientation.

Location	Fenestration orientation		RM‐ANOVA
	Horizontal (%) (*n* = 3)	Non‐horizontal (%) (*n* = 4)	*F* statistic (*p*‐value)
Central	0.49 ± 0.70	0.26 ± 0.21	Orientation × location 5.81 (0.02) Post hoc comparison: Peripheral: H < N (0.01)
Mid‐peripheral	0.00 ± 1.90	1.21 ± 0.34	
Peripheral	−2.92 ± 0.63	2.49 ± 1.03	

## DISCUSSION

The key finding from the current study was that a single peripheral fenestration significantly reduced corneal oedema in healthy eyes when averaged across all corneal locations compared to non‐fenestrated lenses, after correcting for variations in fluid reservoir thickness. This reduction in oedema could potentially be attributed to improved tear exchange facilitated by the fenestration (either via the fenestration or the landing zone), resulting in greater oxygen delivery and enabling the removal of metabolic by‐products.

Interestingly, the reduction in oedema with fenestrated lenses was not uniform across all corneal locations. The peripheral cornea exhibited the greatest difference in oedema with −0.15 ± 0.98% for the fenestrated lenses, compared to 1.81 ± 0.57% for the non‐fenestrated lenses. This effect was greater in the periphery when considering the participants with the fenestration located along the horizontal meridian, aligned with the orientation of the OCT scan (−2.04 ± 1.06% for the fenestrated lenses compared to 3.34 ± 1.50% for the non‐fenestrated lenses). This suggests that a peripheral fenestration has a more pronounced effect on the peripheral cornea, consistent with previous studies which indicated that fenestrations have a local effect on corneal oedema. For example, in soft contact lens studies, a large 3–5 mm central fenestration virtually eliminated central oedema,^25^ and small peripheral fenestrations (4 × 0.8 mm diameter) reduced peripheral oedema by one‐third.^1^ Hill et al.^19^, ^20^, ^26^ also demonstrated that, for corneal rigid lenses, changes in epithelial oxygen uptake were only observed within ~1 mm of fenestrations, suggesting a localised effect. The peripheral cornea, being closer to the limbus where the fenestration was positioned, potentially experienced increased tear exchange either directly through the fenestration or via the landing zone (due to reduced suction pressure) or greater passive diffusion of oxygen through the fenestration.

The effect of fenestration orientation upon corneal oedema was also examined to further investigate the localised reduction in corneal oedema. Since OCT scans were only obtained along the horizontal meridian, if the fenestration produced a local reduction in oedema, participants with the fenestration located along the horizontal meridian would be expected to exhibit a greater reduction in oedema relative to the central location compared to participants with the fenestration oriented at a non‐horizontal meridian. The data supported this hypothesis (Table 4, Figure 5), with participants with the fenestration located along the horizontal meridian displaying significantly less relative peripheral oedema (−2.92 ± 0.63%) compared to the participants with the fenestration located along a non‐horizontal meridian (2.49 ± 1.03%), which also supports a localised response. Participants with the fenestration positioned along the horizontal meridian may have experienced less peripheral oedema because the fenestration was not covered by the eyelids throughout lens wear, facilitating greater exposure to atmospheric oxygen. Other studies have also reported less corneal oedema along the continuously exposed horizontal corneal meridian compared to the vertical meridian, which is partially covered by the eyelids during open‐eye lens wear.^27^

The findings of the current study have implications for the design and fitting of scleral lenses in eyes with a greater risk of corneal oedema, such as post‐graft, post‐radial keratotomy or older eyes with reduced endothelial function. Incorporating fenestrations into scleral lens designs could be a viable strategy to enhance tear exchange and peripheral corneal oxygenation, thereby reducing the risk of peripheral corneal oedema. However, the orientation and potentially the size and number of the fenestrations are important factors that should be considered during lens design and fitting. Scleral lens fenestrations may also offer additional benefits, including reduced fluid reservoir debris and midday fogging, easier lens removal due to reduced suction forces, and less conjunctival compression and limbal redness.^18^ However, an increased number or larger size of fenestrations could lead to potential drawbacks, such as greater fluid reservoir turbidity or conjunctival prolapse through the fenestration.

This study also raises interesting questions about the potential long‐term effects of fenestrated scleral lenses on corneal health. While the localised reduction in peripheral oedema following short‐term lens wear observed in the current study is promising, further research is needed to evaluate the long‐term impact of fenestrated lenses on corneal physiology, particularly in patients with compromised corneal health in a large clinical cohort. The limitations of the current study were primarily the small sample size, the inclusion of young healthy adults with normal corneas and the short duration of lens wear. Variations in fluid reservoir thickness between the lens conditions were a potential confounder, but were corrected using corneal response data from the same participants when varying fluid reservoir thickness in a prior experiment.^23^ Small variations in lens thickness were also observed between the two lens types; however, the 10‐μm difference in peripheral lens thickness would likely yield around a 0.01% difference in corneal oedema (based on previous work using high Dk materials^10^) and would not alter the 2% difference in peripheral oedema observed between the fenestrated and non‐fenestrated lenses (Table 3).

## CONCLUSION

In conclusion, the present study provides evidence that a single peripheral fenestration can significantly reduce scleral lens‐induced peripheral corneal oedema in healthy eyes when controlling for lens and fluid reservoir thickness. This finding has implications for the design and fitting of scleral lenses, particularly for eyes at risk of corneal oedema.

## AUTHOR CONTRIBUTIONS

**Asif Iqbal:** Conceptualization (supporting); data curation (supporting); formal analysis (supporting); investigation (supporting); methodology (supporting); project administration (supporting); resources (supporting); visualization (supporting); writing – original draft (lead); writing – review and editing (lead). **Damien Fisher:** Conceptualization (lead); data curation (lead); formal analysis (supporting); investigation (lead); methodology (lead); project administration (lead); resources (lead); supervision (supporting); visualization (lead); writing – original draft (supporting); writing – review and editing (supporting). **David Alonso‐Caneiro:** Formal analysis (supporting); investigation (supporting); methodology (supporting); project administration (supporting); resources (supporting); supervision (supporting); visualization (supporting); writing – review and editing (supporting). **Michael J. Collins:** Conceptualization (lead); data curation (supporting); formal analysis (supporting); investigation (supporting); methodology (supporting); project administration (supporting); resources (supporting); supervision (supporting); visualization (supporting); writing – review and editing (supporting). **Stephen J. Vincent:** Conceptualization (lead); data curation (supporting); formal analysis (lead); investigation (supporting); methodology (supporting); project administration (supporting); resources (lead); supervision (lead); visualization (lead); writing – original draft (lead); writing – review and editing (lead).

## FUNDING INFORMATION

Asif Iqbal and Damien Fisher were funded by a QUT Postgraduate Research Award PhD scholarship and a Dorothy Carlborg Research Award from the Cornea and Contact Lens Society of Australia.

## CONFLICT OF INTEREST STATEMENT

The authors report no conflicts of interest and have no proprietary interest in any of the materials mentioned in this article.
